# Synthesis of Li_2_Ti_3_O_7_ Anode Materials by Ultrasonic Spray Pyrolysis and Their Electrochemical Properties

**DOI:** 10.3390/ma6062285

**Published:** 2013-06-03

**Authors:** Takashi Ogihara, Takayuki Kodera

**Affiliations:** Graduate School of Material Science and Engineering, University of Fukui, 3-9-1 Bunkyo, Fukui 910-8507, Japan; E-Mail: t-kodera@u-fukui.ac.jp

**Keywords:** spray pyrolysis, lithium ion battery, powders, anode, oxide

## Abstract

Ramsdellite-type lithium titanate (Li_2_Ti_3_O_7_) powders were synthesized by performing ultrasonic spray pyrolysis, and their chemical and physical properties were characterized by performing Scanning Electron Microscope (SEM), powder X-ray Diffraction (XRD), and Inductively Coupled Plasma (ICP) analyses. The as-prepared Li_2_Ti_3_O_7_ precursor powders had spherical morphologies with hollow microstructures, but an irregularly shaped morphology was obtained after calcination above 900 °C. The ramsdellite Li_2_Ti_3_O_7_ crystal phase was obtained after the calcination at 1100 °C under an argon/hydrogen atmosphere. The first rechargeable capacity of the Li_2_Ti_3_O_7_ anode material was 168 mAh/g at 0.1 C and 82 mAh/g at 20 C, and the discharge capacity retention ratio was 99% at 1 C after the 500th cycle. The cycle performance of the Li_2_Ti_3_O_7_ anode was also highly stable at 50 °C, demonstrating the superiority of Li_2_Ti_3_O_7_ anode materials reported previously.

## 1. Introduction

Lithium ion batteries (LIBs) are expected to be used for energy storage in electric vehicles (EVs) as well as for load leveling for photovoltaic power generation or wind power generation. Until now, carbon has usually been used as an anode material for these LIBs [1,2,3]. However, a solid-electrolyte interface layer usually forms on carbon anodes at a potential below 0.8 V, which leads to active lithium loss over time resulting in increases in impedance, decreases in the rechargeable capacity, and ultimately shorter life cycles of the LIBs. For the LIBs in EVs, oxide-type materials are candidates for the anode because of their better safety performance and long life cycles. Spinel-type Li_4_Ti_5_O_12_ (denoted as LTO) [4,5] has been investigated as an alternative anode material and was found to have a long plateau at 1.5 V and an excellent life cycle due to its structural stability during the Li ion insertion process. However, the disadvantage of LTO as an anode material is the low electronic conductivity resulting from its insulating ionic crystal structure [6]. To improve its electric conductivity, carbon is usually added to LTO, and this has been relatively effective in improving all its electrochemical properties. In particular, it is well known that solution techniques, such as the sol-gel method, spray drying, and spray pyrolysis make it possible to homogeneously dope the LTO powders with carbon [7,8,9]. On the other hand, ramsdellite-type Li_2_Ti_3_O_7_ (denoted as RLTO) already has good electrical conductivity [10]. Therefore, it can be used as an anode without the need for carbon doping. Furthermore, the electrochemical properties of RLTO anodes, prepared using the solid-state reaction have already been reported [11,12,13].

In this paper, the powder characteristics and electrochemical properties of RLTO anode materials synthesized using ultrasonic spray pyrolysis were described in detail. In the spray pyrolysis, the electrochemical properties of RLTO anode materials, such as their rechargeable capacity, life cycle, and rate performance, have not been reported. It is well known that spray pyrolysis easily and quickly leads to the formation of homogeneous double oxide powders in one step. In fact, various types of cathode materials for LIBs [14,15,16,17] such as LiMn_2_O_4_, LiNi_0.5_Mn_1.5_O_4_, LiNi_1/3_Mn_2/3_O_4_, and LiFePO_4_ have been prepared previously using spray pyrolysis. It was reported that their powder characteristics such as the particle size, morphology, and chemical composition were controlled well, and excellent recharging performance was demonstrated. In this work, it is expected that the recharging properties of RLTO anode materials derived from spray pyrolysis will be even better.

## 2. Results and Discussion

**Figure 1:**
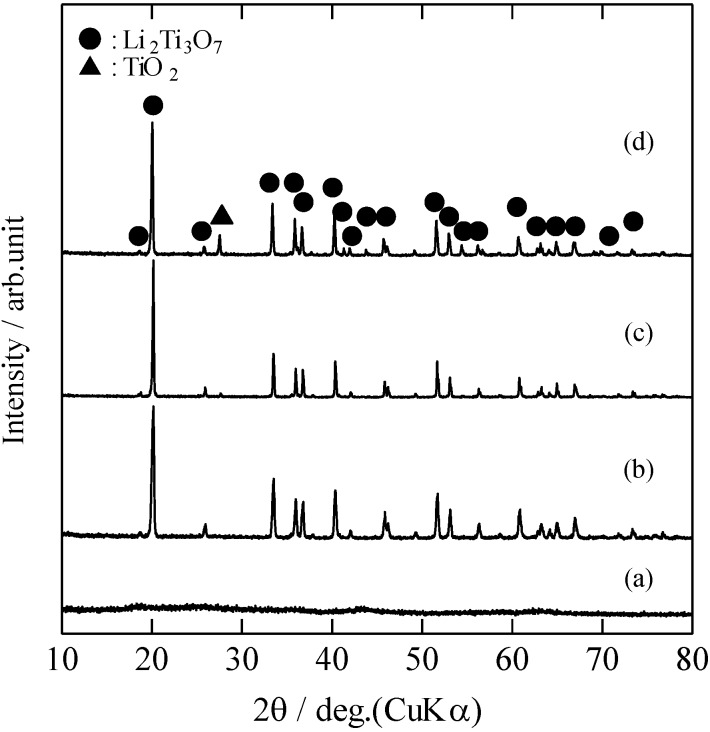
XRD pattern of (**a**) as-prepared powders; and (**b**) ramsdellite-type Li_2_Ti_3_O_7_ (RLTO) powders calcined at 1100 °C under argon/hydrogen; (**c**) air; (**d**) nitrogen.

**Figure 2:**
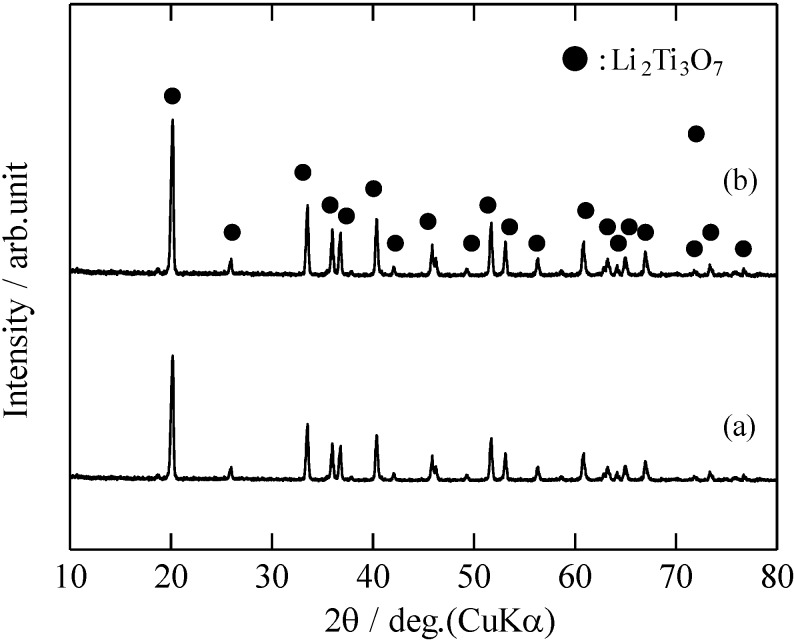
XRD pattern of RLTO powders calcined at 1100 °C for 24 h under (**a**) air; and (**b**) nitrogen.

Figure 1 shows the XRD patterns of the RLTO precursor powders and RLTO powders calcined at 1100 °C under nitrogen, air, and argon/hydrogen atmospheres. The crystal phase of the RLTO precursor powders was amorphous, as shown in Figure 1a. Therefore, it can be assumed that the Li_2_O and TiO_2_ remained amorphous in the precursor particles. The XRD pattern of the RLTO powder calcined under argon/hydrogen atmosphere was in good agreement with that of the ramsdellite phase (space group: *Pbnm*), and no other phases were observed. On the other hand, a small amount of the rutile phase (space group: *P*42/*mnm*) was observed in RLTO powders calcined under air and nitrogen atmospheres. Figure 2 shows the XRD patterns of the RLTO powders calcined at 1100 °C for 24 h under the nitrogen and air atmosphere. It was found that the single phase of RLTO was formed by the calcination for 24 h. Table 1 shows the chemical composition of the as-prepared powders and RLTO powders calcined at 1100 °C as determined by inductively coupled plasma (ICP) analysis. The chemical composition of the as-prepared RLTO powders and those calcined at 1100°C was in agreement with the starting solution composition. This suggests that the Li^+^ and Ti^4+^ ions were homogeneously blended in each mist. Thus, it is confirmed that the spray pyrolysis technique yielded homogeneous RLTO anode materials.

**Table 1:** Chemical compositions of as-prepared powders and RLTO powders calcined at 1100 °C.

Sample	Atomic conc. (molar ratio)	
	Li	Ti
As-prepared powders	1.02	1.488
RLTO powders ^a^	1.01	1.489

^a^ Calcination at 1100 °C under argon/hydrogen atmosphere.

**Figure 3:**
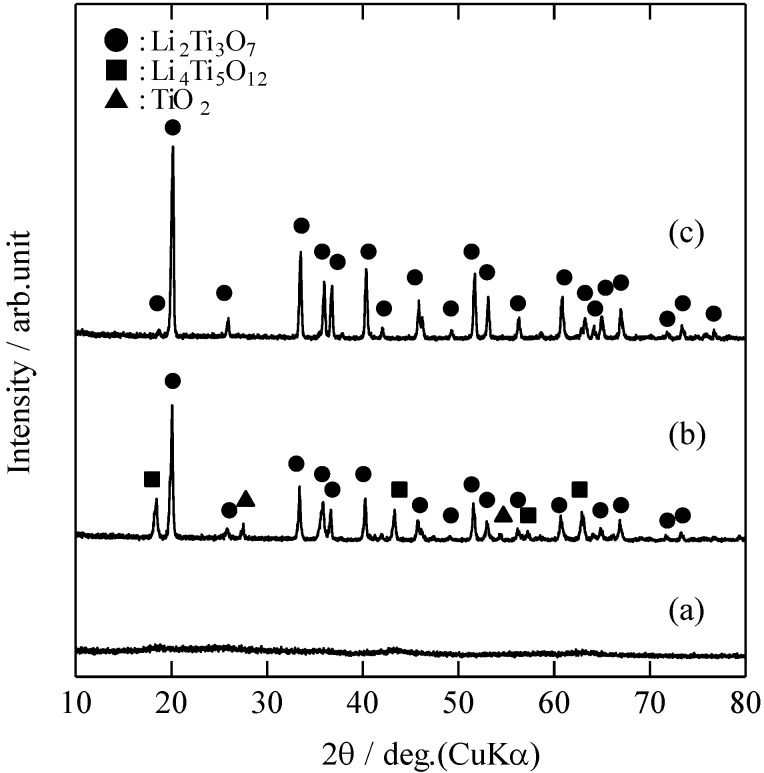
XRD pattern of (**a**) as-prepared powders and RLTO powders calcined under argon/hydrogen; at (**b**) 800 °C; and (**c**) 1000 °C.

Figure 3 shows the XRD patterns of the RLTO precursor powders and the RLTO powders calcined under the argon/hydrogen atmosphere at different temperatures. Spinel-type LTO and rutile-type TiO_2_ were observed in addition to the RLTO phase for the powder calcined at 800 °C. This indicates that the reaction between Li_2_O and TiO_2_ in the precursor particles is influenced by the atmosphere and temperature during calcination. In particular, this reaction was promoted by a reduction atmosphere.

**Figure 4:**
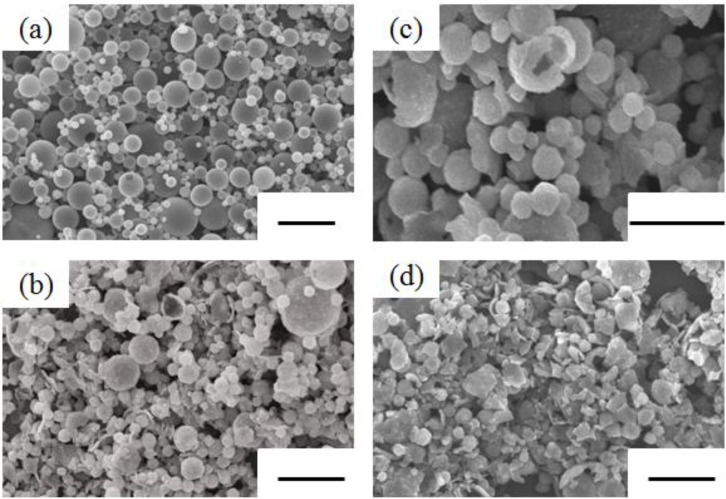
SEM photographs of (**a**) as-prepared powders and RLTO powders calcined at (**b**) 800 °C; (**c**) 900 °C; and (**d**) 1100 °C (bar = 5 μm).

Figure 4 shows typical SEM photographs of the as-prepared powders and the RLTO powders. The concentration of starting solution was 0.25 M. The RLTO precursor powder particles had a spherical morphology with an average diameter of 1.4 μm. The geometric standard deviation of the average particle size was 1.4. This suggested that 68% of all particles ranged from 0.56 µm to 1.96 µm. Therefore, it was found that the as-prepared powders had a relatively broad particle size distribution. This resulted in a large droplet size distribution in the mist derived from the piezoelectric transducer. Furthermore, hollow particles were obtained. It is believed that the pressure of the mist inside these hollow particles was increased by the intense combustion of citric acid during the formation of the RLTO particles, which caused them to break out of their shells. The average particle size increased from 1 μm to 1.8 μm when the concentration of starting solution was increased from 0.1 M to 0.5 M. On the other hand, the geometrical standard deviation was independent of the concentration of starting solution. It has been reported that the droplet size distribution of mist was not affected by the concentration in the spray pyrolysis [18,19,20]. The average particle size decreased from 1.6 μm to 1.4 μm when the pyrolysis temperature was increased from 500 °C to 800 °C. The average particle size was increased with the concentration of starting solution, but independent of the pyrolysis temperature. The RLTO particles also gradually changed to irregular shapes as the calcination temperature was increased. This suggests that the RLTO particles derived from spray pyrolysis have high sinterability. So far, it has been reported that the oxide powders derived from spray pyrolysis consisted of the primary particles with nano-size order and had a high sinterability [21,22].

The specific surface area of RLTO precursor powders determined by BET method was 50 m^2^/g. This suggested that the RLTO precursor powders consisted of the primary particles. After calcination at 1100 °C, the specific surface area of RLTO powders decreased to 24 m^2^/g. It was considered that the primary particles were rapidly sintered by the calcination.

**Figure 5:**
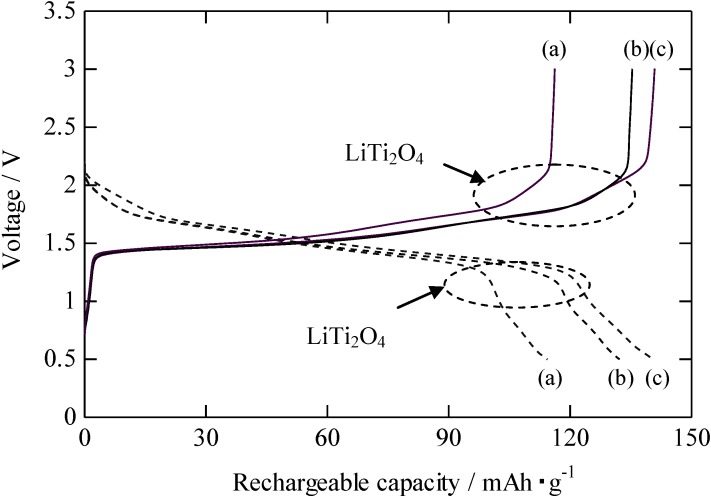
Rechargeable curves of RLTO anode material calcined at 1100 °C under (**a**) nitrogen; (**b**) air; and (**c**) argon/hydrogen.

Figure 5 shows the first recharging curves obtained for the RLTO anode material at 25 °C. The charge and discharge rate was 1 C. A typical long plateau attributed to the reduction/oxidation of the Ti^4+^/Ti^3+^ couple in the RLTO was observed at approximately 1.5 V in all recharging curves. The first discharge capacities at 1 C of the RLTO anodes calcined under nitrogen, air, and argon/hydrogen atmospheres were 114 mAh/g, 135 mAh/g, and 140 mAh/g, respectively. Thus, the rechargeable capacity of RLTO anode calcined under argon/hydrogen was the highest. This indicates that the reduction atmosphere in the argon/hydrogen easily led to the formation of Ti^4+^/Ti^3+^ couple, compared to nitrogen and air.

A short plateau was also observed at 2.3 V during the charging and at 1.3 V during the discharging, which can be attributed to the formation of LiTi_2_O_4_. Gover *et al*. [9] reported a short plateau in the recharging curves at the same voltage and concluded that rechargeable LiTi_2_O_4_ existed in the RLTO. Therefore, although small-sized TiO_2_ crystals were not detected by XRD, they remained in the RLTO powders calcined under argon/hydrogen atmosphere. In XRD and ICP analysis, it was confirmed that the RLTO powder had a homogeneous composition. However, the existence of TiO_2_ was detected in the electrochemical measurement. It was considered that RLTO powder had inhomogeneous composition at the molecular level.

**Figure 6:**
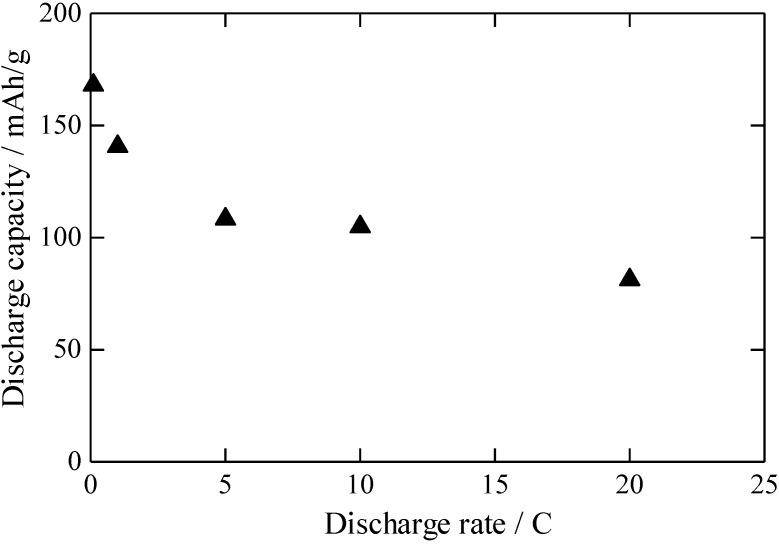
Relation between discharge rate and discharge capacity of RLTO anode material calcined at 1100 °C under argon/hydrogen.

Figure 6 shows the relation between the discharge capacity and the discharge rate of the RLTO anode material calcined under an argon/hydrogen atmosphere. The charge rate was equal to the discharge rate given at each plot. The discharge capacity of the RLTO anode was 168 mAh/g at 0.1 C, which is partially due to its homogeneous crystal phase as discussed above. The discharge capacity of the RLTO anode decreased to 83 mAh/g when the discharge rate was increased to 20 C, probably because of the presence of LiTi_2_O_4_. Nevertheless, it has not been reported previously that the discharge capacity of RLTO is around 80 mAh/g at 20 C.

**Figure 7:**
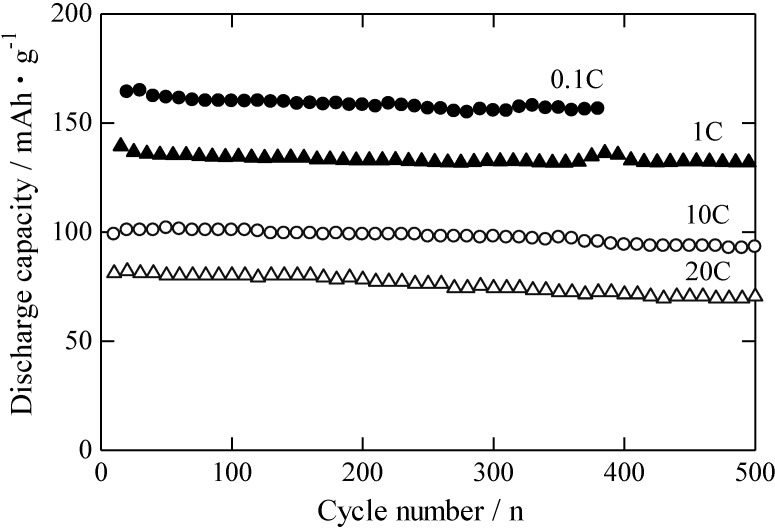
Relation between cycle number and discharge capacity of RLTO anode material calcined at 1100 °C under argon/hydrogen.

Figure 7 shows the relation between the cycle number and the discharge capacity of the RLTO anode material calcined under an argon/hydrogen atmosphere at various discharge rates. The recharging process was cycled up to 500 times at 25 °C. The charge rate was equal to the discharge rate indicated in Figure 7. The RLTO anode material clearly had excellent cycle stability, similar to that of spinel-type LTO. In particular, the discharge capacity remained over 90% of the initial value after 100 cycles, probably because the structure of RLTO was highly stable during the intercalation of Li^+^ ions in the recharging process. This suggests that RLTO has a high potential as an anode material for LIBs in EVs or for load leveling for photovoltaic power generation or wind power generation.

**Figure 8:**
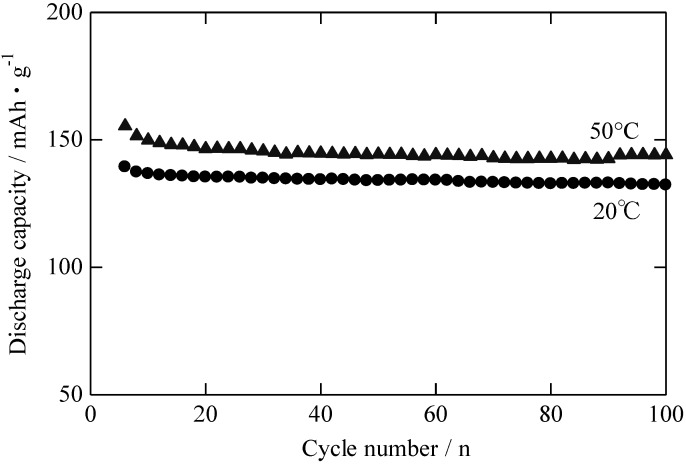
Cycle stability of RLTO anode material at 25 °C and 50 °C.

Figure 8 shows the relation between the cycle number and discharge capacity of the RLTO anode material measured at 25 °C and 50 °C, respectively. The charge and discharge rate was 1 C, respectively. The first discharge capacities of the RLTO anode material at 25 ºC and 50 °C were 135 mAh/g and 155 mAh/g, respectively. This suggests that the electric conductivity of RLTO at 50 °C is higher than that of RLTO at 25 °C. After 100 cycles at 50 °C, the discharge capacity of the RLTO anode remained above 90% of the initial value.

## 3. Experimental Section

LiNO_3_, Ti(*iso*-OC_3_H_7_)_4_ (denoted as TTIP), and citric acid were used as the starting reagents. First, TTIP was dissolved in 1 M citric acid solution, and then LiNO_3_ was added. The molar ratio of Li/Ti was 2/3 in the citric acid solution, and their total concentration was 0.25 M in the citric acid solution. A mist of the starting solution was generated by the piezoelectric transducer (1.6 MHz) at 25 °C, and this mist was introduced into two electrical furnaces through an alumina tube (38 mm φ × 1000 mm) by an air carrier gas flowing at 10 dm^3^/min. The temperature of the electrical furnace used to dry the mist was set at 400 °C, and that of the furnace used to pyrolyze the mist was set at 650 °C. The as-prepared powders were collected by a cyclone and then calcined from 800 °C to 1100 °C for 2 h under air, nitrogen, or argon/hydrogen (5%) atmospheres.

The crystal phases of the as-prepared and calcined powders were identified by measuring their powder X-ray diffraction (XRD, Shimadzu, XRD-6100, Kyoto, Japan) patterns. The morphology and microstructure of the as-prepared and calcined powders were determined with a scanning electron microscope (SEM, JEOL, JSM-6390, Tokyo, Japan). The specific surface area the as-prepared and calcined powders was measured by BET method with the adsorption of nitrogen gas (BEL Japan Inc., BELSORP-miniII, Osaka, Japan). The electrochemical properties were examined using a coin cell (Hosen Co., Ltd., CR2032, Osaka, Japan). The RLTO powders, acetylene black, and a polyvinylidene difluoride powder were mixed in a weight ratio of 80:10:10 in *N*-methyl-2-pyrrolidone to prepare the slurry. The slurry was coated on an aluminum foil using a doctor blade and dried under vacuum at 100 °C for 24 h. A lithium sheet was used as the counter electrode, a polypropylene sheet (Hosen Co., Ltd., Celgard 2400, Osaka, Japan) was used as a separator, and 1.0 M LiPF_6_ in ethylene carbonate/1,2-dimethoxyethane (EC:DEC = 1:1) was used as the electrolyte. The coin cell was built in a glove box under an argon atmosphere. The rechargeable capacity and cycle stability of the RLTO anode materials were measured with a battery tester (Hosen Co., Ltd., BTS2004, Osaka, Japan) between 0.5 V and 3.0 V. The current density ranged from 0.5 (1 C) to 10 (20 C) mA/cm^2^.

## 4. Conclusions

RLTO powders were successfully synthesized using ultrasonic spray pyrolysis. The as-prepared particles had a spherical morphology with a hollow microstructure, and the final crystal phase varied depending on the calcination atmosphere. The desired RLTO crystal phase was obtained after calcination at 1100 °C under an argon/hydrogen atmosphere. The electrochemical measurement performed using the RLTO anode revealed that the first rechargeable capacities of the RLTO anode were 168 mAh/g and 82 mAh/g at 0.1 C and 20 C, respectively. Furthermore, the RLTO anode had excellent cycle stability at 25 °C and 50 °C, demonstrating the superiority of RLTO anode, as previously reported.
